# Clinical manifestations of reported Lyme disease cases in Ontario, Canada: 2005–2014

**DOI:** 10.1371/journal.pone.0198509

**Published:** 2018-06-01

**Authors:** Karen O. Johnson, Mark P. Nelder, Curtis Russell, Ye Li, Tina Badiani, Beate Sander, Douglas Sider, Samir N. Patel

**Affiliations:** 1 Enteric, Zoonotic and Vector-borne Diseases, Communicable Diseases, Emergency Preparedness and Response, Public Health Ontario, Toronto, Ontario, Canada; 2 Analytic Services, Knowledge Services, Public Health Ontario, Toronto, Ontario, Canada; 3 Dalla Lana School of Public Health, University of Toronto, Toronto, Ontario, Canada; 4 University Health Network, Toronto General Hospital, Toronto, Ontario, Canada; 5 Institute of Health Policy, Management and Evaluation, University of Toronto, Toronto, Ontario, Canada; 6 Institute for Clinical Evaluative Sciences, Toronto, Ontario, Canada; 7 Department of Health Research Methods, Evidence and Impact, McMaster University, Hamilton, Ontario, Canada; 8 Public Health Ontario Laboratory, Public Health Ontario, Toronto, Ontario, Canada; 9 Department of Laboratory Medicine and Pathobiology, University of Toronto, Toronto, Ontario, Canada; University of North Dakota School of Medicine and Health Sciences, UNITED STATES

## Abstract

Lyme disease (LD) is the most common vector-borne disease in Ontario, Canada. We describe the epidemiology and clinical manifestations of LD in Ontario and examine trends in the incidence of non-disseminated and disseminated LD. LD surveillance data from the integrated Public Health Information System (iPHIS) from 2005–2014 were mapped to symptoms according to syndrome groups (erythema migrans (EM), flu-like, cardiac, neurologic or arthritic) and disease stages (early localized, early disseminated or late disseminated). During the study period, 1,230 cases due to *Borrelia burgdoferi* were reported in Ontario with annual incidence rates ranging from 0.32 (2006) to 2.16 (2013) cases per 100,000 population. Seventy percent of cases had EM and the proportion of cases with EM increased over time. Other clinical manifestations included flu-like (75%), arthritic (42%), neurologic (41%) and cardiac (6%) symptoms. Early localized disease (n = 415) manifested with EM (87%) and flu-like (57%) symptoms; early disseminated disease (n = 216) manifested with neurologic (94%), cardiac (10%) and EM (63%) symptoms; and late disseminated disease (n = 475) manifested with EM (62%), neurologic (55%), cardiac (9%), and arthritic symptoms (i.e., arthralgia (93%) and arthritis (7%)). Early localized and early disseminated cases (88% each) occurred primarily from May through September, compared to late disseminated cases (81%). The proportion of cases reported to public health within 30 days of illness onset increased during the study period, while the proportion of cases reported within 1–3 months and >3 months decreased. Geographical variations characterized by higher incidence of early localized disease and earlier public health notification (within 30 days of illness onset) occurred in regions with established or recently established LD risk areas, while later public health notification (>3 months after illness onset) was reported more frequently in regions with recently established or no identified risk areas. This is the first study to describe the clinical manifestations of LD in Ontario, Canada. The observed geographical variations in the epidemiology of LD in Ontario reinforce the need for regionally focused public health strategies aimed at increasing awareness, promoting earlier recognition and reporting, and encouraging greater uptake of preventive measures.

## Introduction

Infection with *Borrelia burgdoferi* results in a multi-system disease that is characterized by three clinically defined stages: early localized, early disseminated and late disseminated Lyme disease (LD). Erythema migrans (EM) is the most common manifestation of early localized LD, with up to 80% of infected persons presenting with either the typical bull’s eye rash or an atypical rash without central clearing [1–3]. EM rash occurs two to 30 days after the tick bite and may be accompanied by flu-like illness that includes fever, chills, myalgia, arthralgia and stiff neck. Secondary EM rashes remote to the initial site of EM occur in 50% of cases [1] and are one of the earliest indications of early disseminated LD, which typically occurs weeks to months after an untreated infection. Early disseminated disease characterized by neurological manifestations including headache, stiff neck, pain or tingling in the extremities, Bell’s palsy, mood disorders, memory deficits and sleep disorders develops in 15–20% [4] of untreated cases, while Lyme carditis, frequently presenting as atrioventricular block, occurs in 1% of untreated cases [5]. The most common manifestation of late disseminated LD is arthritis, which appears weeks to years after initial infection in up to 50% of people with untreated infection [6, 7]. Lyme arthritis is characteristically intermittent and mainly affects large joints including the shoulders and knees, with pain, effusion and synovitis being the typical presentations.

Since 2007, LD has emerged as the most important vector-borne disease in the province of Ontario, Canada, accounting for the majority of cases reported nationally. In Canada, available public health and vector surveillance data have largely focused on identifying geographically defined risk areas and describing the epidemiology of the disease. A minimal number of studies in Canada, and no known studies in Ontario have been published on the clinical manifestations of LD. Two recent studies described the clinical manifestations of LD in the province of Nova Scotia [8] and in Canada overall [9]. However, these studies did not examine the relationships between clinical stages of LD and seasonality, regionalization, time to public health notification or clinical outcomes such as hospitalization. The current study aims to fill this gap by describing the epidemiology and clinical manifestations of LD due to *B*. *burgdorferi* in Ontario and examining trends in the incidence of non-disseminated and disseminated LD using data obtained from the provincial reportable diseases surveillance system. The findings from our study may serve as a source of information for the development and implementation of targeted evidenced-based diagnostic, prevention and control strategies.

## Materials and methods

### Case data

LD cases are reported to the province by 36 health units through the web-based integrated Public Health Information System (iPHIS). Health units are responsible for case management including the classification of cases according to case definitions in use at the time of notification, and for collecting information on demographics, exposures, symptoms and outcomes. Data on LD cases with episode dates from 2005–14 were extracted from iPHIS. Under the revised case definitions that came into effect in 2009, a probable designation was assigned to certain cases previously reported as confirmed. To ensure comparability, cases reported from 2005–08 are based on confirmed counts only, while those from 2009–14 are based on the sum of confirmed and probable counts. The surveillance case definitions used during the study period can be found in S1 File.

### Symptoms

Symptoms, self-reported by cases and/or reported by clinicians as part of public health investigations, were classified into three mutually exclusive disease stages by an infectious diseases physician and an epidemiologist following a review of LD symptoms reported in the literature [1–5, 7, 10, 11]. Reported symptoms were first mapped to syndrome groups (e.g. EM, flu-like, cardiac, neurologic or arthritic) and then to disease stages (S1 Table). Cases with or without EM and/or non-specific symptoms without any systemic manifestations were classified as early localized disease; cases with symptoms consistent with Lyme carditis and/or neuroborreliosis with or without EM and/or non-specific symptoms were classified as early disseminated disease; and cases with any arthritic manifestations were classified as late disseminated disease. Cases that reported no symptoms or symptoms that could not be classified were excluded from specific analyses.

### Time to public health notification

Time to notification was calculated as the elapsed time between symptom onset and public health notification of the case. Time to notification was categorized as notifications occurring in ≤30 days, within 1–3 months or >3 months from symptom onset based on the respective estimates of the range of onsets for early localized disease, and early and late disseminated disease, if an earlier stage was untreated [12].

### Other variables

Age, sex, health unit region of residence, episode date, classification (i.e., confirmed or probable) and hospitalization status were included in analyses.

### Statistical analyses

We calculated annual number of LD cases and disease stage specific incidence rates per 100,000 population using population estimates obtained from Statistics Canada [13]. Changes in incidence over time, and between syndrome groups were evaluated using Poisson regression with case counts as the response and population as the offset. Between group differences in demographic and clinical measures were assessed. All analyses were completed using SAS version 9.3 (SAS Institute Inc., Cary, NC, US); p values < 0.05 were considered statistically significant for all analyses.

### Ethics statement

iPHIS data are routinely collected for surveillance and epidemiologic purposes and the extract used for this study did not include personal identifiers. As a result, ethics approval was not required.

## Results

### Epidemiology—All cases

#### Time trend

**Fig 1:**
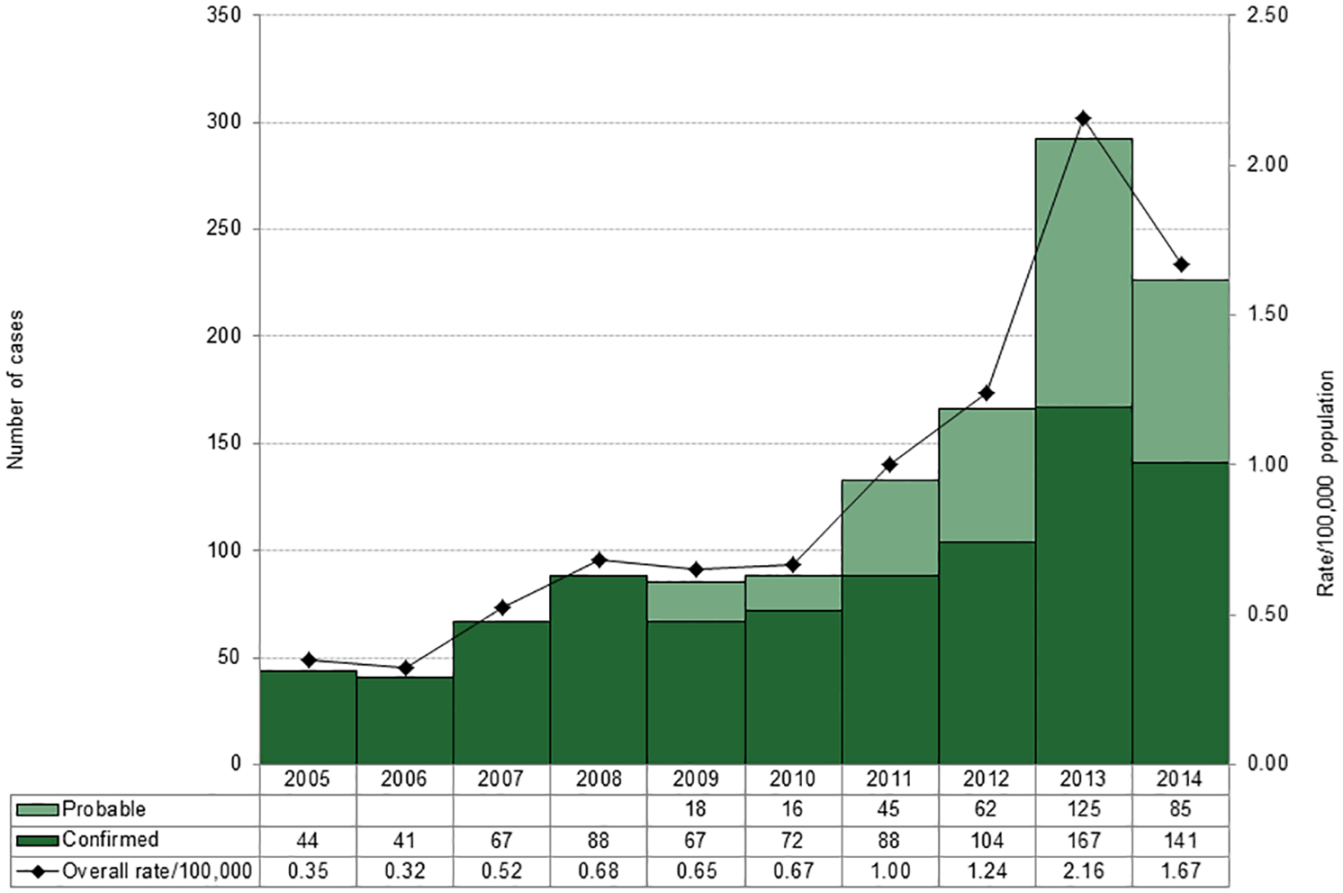
Number and incidence rate of Lyme disease cases by classification, Ontario: 2005–14 (n = 1230)*. * Probable cases are included after 2009 as they are equivalent to a subset of confirmed cases reported in prior years.

During the period 2005–14, 1,252 cases of LD were reported in Ontario. The majority of cases were due to *B*. *burgdorferi* (n = 1,230), followed by *B*. *afzelii* (n = 21) and *B*. *garinii* (n = 1). Cases caused by *B*. *afzelii* and *B*. *garinii* were excluded from further analyses as these agents are not endemic in North America. The annual number of cases due to *B*. *burgdorferi* varied from a low of 41 in 2006 to a high of 292 in 2013; corresponding incidence rates ranged from 0.32 to 2.16 cases per 100,000 population (Fig 1). Over the period, the annual number of cases increased more than five-fold. Since 2009, a probable case definition has been in use and this classification accounted for 18.2–42.8% of LD cases reported annually from 2009–14. Table 1 shows that there were significant differences in the epidemiology and clinical characteristics of the three studied LD stages with respect to mean time to public health notification (p < 0.0001), region of residency (p = 0.0026; data not shown), having illness onset from May to September (p = 0.013), hospitalization status (p = 0.0274) and presentation of EM and flu-like symptoms (p < 0.0001). No significant age or sex differences by stage were observed.

**Table 1:** Characteristics of Lyme disease cases by stage, Ontario: 2015–14.

Disease stage	Early localized	Early disseminated	Late disseminated	All Lyme disease cases*	P value
**Incidence**					
Number of cases (%)	415 (36.6)	216 (19.1)	475 (41.9)	1230 (100)	n/a
Rate/100,000	0.32	0.17	0.36	0.94	n/a
**Regions (rate/100,000)**					
Central East	73 (0.19)	40 (0.11)	53 (0.14)	192 (0.51)	n/a
Central West	59 (0.24)	39 (0.16)	62 (0.25)	169 (0.67)	n/a
Eastern	182 (1.06)	89 (0.52)	251 (1.46)	573 (3.33)	n/a
North East	5 (0.09)	0 (0.00)	7 (0.12)	13 (0.23)	n/a
North West	2 (0.08)	4 (0.17)	7 (0.29)	21 (0.88)	n/a
Southwest	16 (0.10)	13 (0.08)	29 (0.18)	67 (0.42)	n/a
Toronto	78 (0.29)	31 (0.12)	66 (0.25)	195 (0.73)	n/a
**Demographic**					
Age range (years)	1–87	1–88	2–93	1–93	n/a
Mean (SD) age (years)	43.7 (21.7)	43.8 (20.0)	43.6 (19.4)	43.7 (20.3)	0.9904^‡^
Median	47.0	46.5	46.0	47.0	
Sex (% male)	52.7	46.3	50.5	50.2	0.3170^#^
**Clinical measures**^†^					
Hospitalization, n (%)	33 (8.0)	32 (14.8)	52 (11.0)	137 (11.1)	0.0274^#^
Erythema migrans, n (%)	362 (87.2)	135 (62.5)	292 (61.5)	789 (69.6)	<0.0001^#^
Flu-like, n (%)	236 (56.9)	190 (88.0)	421 (88.6)	847 (74.8)	<0.0001^#^
Cardiac, n (%)	0 (0%)	22 (10.2)	43 (9.1)	65 (5.7)	<0.0001^#^
Neurologic, n (%)	0 (0%)	204 (94.4)	259 (54.5)	463 (40.9)	<0.0001^#^
Arthritic, n (%)	0 (0%)	0 (0%)	475 (100)	475 (41.9)	<0.0001^#^
**Temporal measures** ^₤^					
Mean (SD) time to notification (days)	43.9 (122.1)	67.4 (147.8)	120.5 (226.9)	86.2 (207.7)	<0.0001^‡^
≤30 days (%)	54.8	44.3	35.5	45.2	<0.0001^#^
1–3 months (%)	34.7	42.5	40.0	38.0	0.1183^#^
>3 months (%)	10.4	13.2	24.5	16.8	<0.0001^#^
Onset May-Sept (%)	87.3	88.2	81.2	84.9	0.0130^#^

* Includes 124 cases for whom disease stage could not be determined due to symptoms that are missing or not related to LD.

^†^ Clinical measures, except hospitalization, include only cases with ≥ 1 reported symptom (n = 1,133).

^₤^Temporal measures include only cases with a symptom onset date (n = 1,104).

^‡^ ANOVA test

^#^ Chi-square test

#### Age and sex

**Fig 2:**
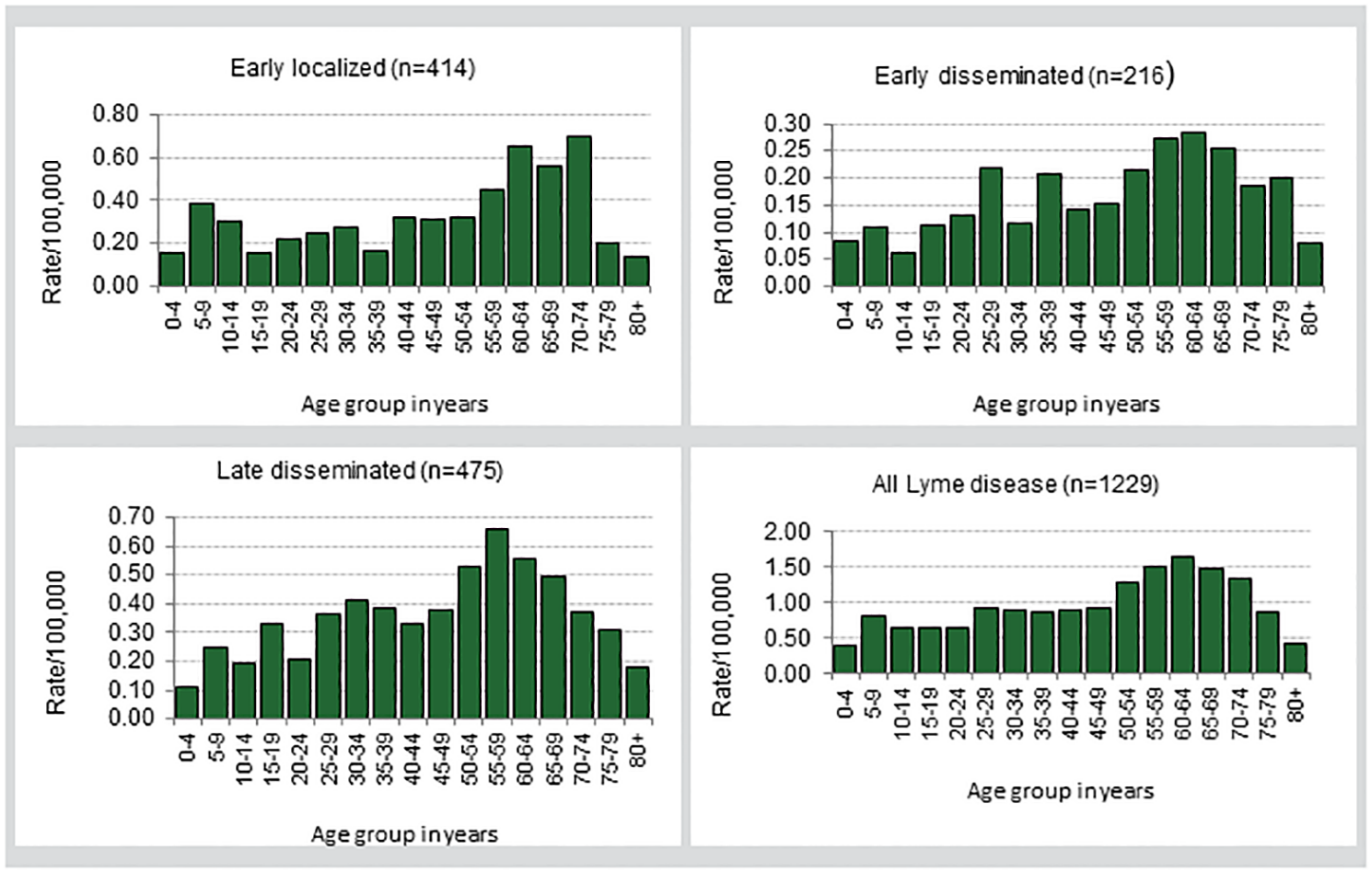
Age-specific incidence rate per 100,000 for Lyme disease cases by clinical stage, Ontario: 2005–2014*^†^. * Ranges for scales are different for each chart. ^†^ All charts do not include cases with missing data on age; chart for all cases includes cases for whom disease stage could not be determined.

Males accounted for 50.2% of reported LD cases (Table 1). Age among the cases ranged from 1–93 years (median = 47.0 years). The highest incidence rates occurred among cases aged 50–74 years (Fig 2).

#### Geographic distribution

**Fig 3:**
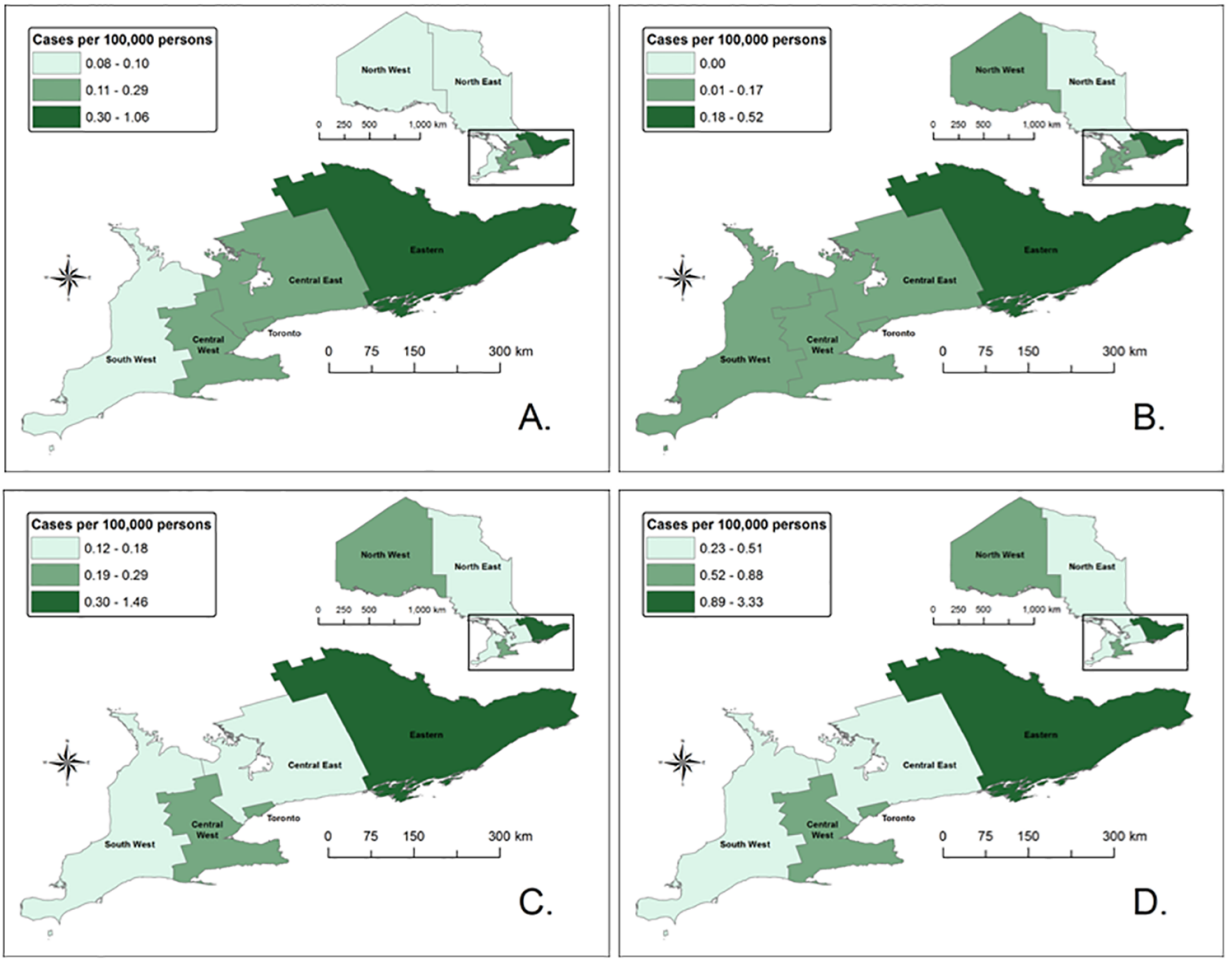
Incidence rates for Lyme disease cases by disease stage and region of residence, Ontario: 2005–14*. * **A** represents early localized LD cases (n = 415), **B** represents early disseminated LD cases (n = 216), **C** represents late disseminated cases (n = 475) and **D** represents all LD cases, including cases with no reported symptoms and cases with symptoms not related to LD (n = 1,230).

Geographic variations in average incidence rates were observed, ranging from 0.23 cases per 100,000 population in North East Region to 3.33 cases per 100,000 population in Eastern Region (Fig 3, Table 1). Overall, annual incidence rates increased for five (i.e., Central East, Central West, Eastern, Southwest and Toronto) of seven regions over the period 2005–14 with the highest incidence rates occurring in health units in Eastern, Central West and North West regions. Tests for trend over time showed that rates of increase in the incidence of LD overall and by stage was fastest in Eastern Region (p < 0.001 for all analyses); however, rates of increase in the other six regions over time and compared to each other were not significant (p > 0.05 for all analyses).

#### Seasonality

**Fig 4:**
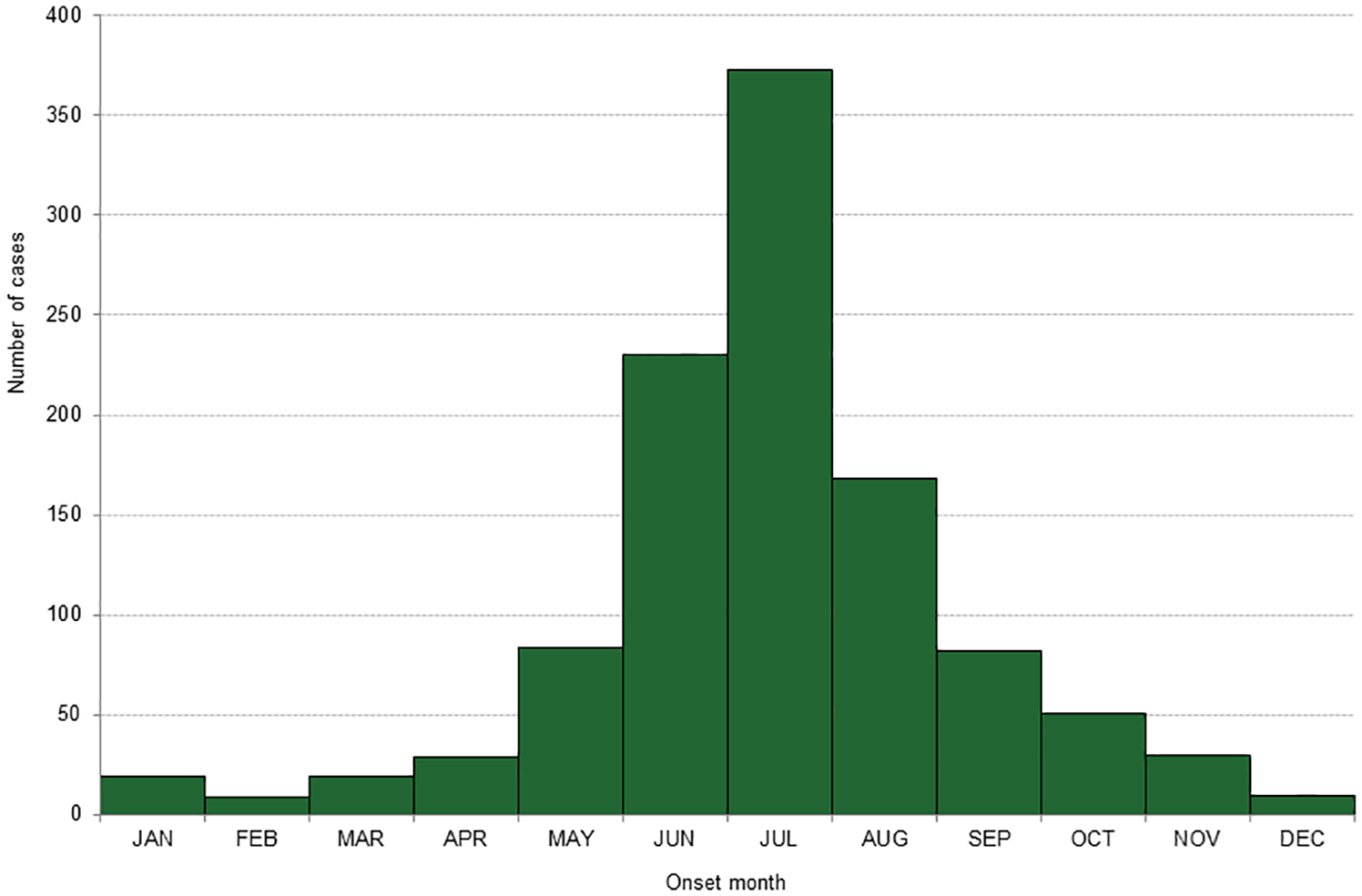
Number of Lyme disease cases by month of symptom onset, Ontario: 2005–14 (n = 1,104)*. * Includes only cases with an onset date for symptoms reported.

LD cases occurred throughout the year with a clear seasonal pattern. Of cases with known dates of onset, 84.9% (937/1,104) occurred from May through September (Fig 4). Early localized (87.3%) and early disseminated (88.2%) cases were more likely to have occurred from May through September compared to late disseminated cases (81.2%).

#### Time to public health notification

**Fig 5:**
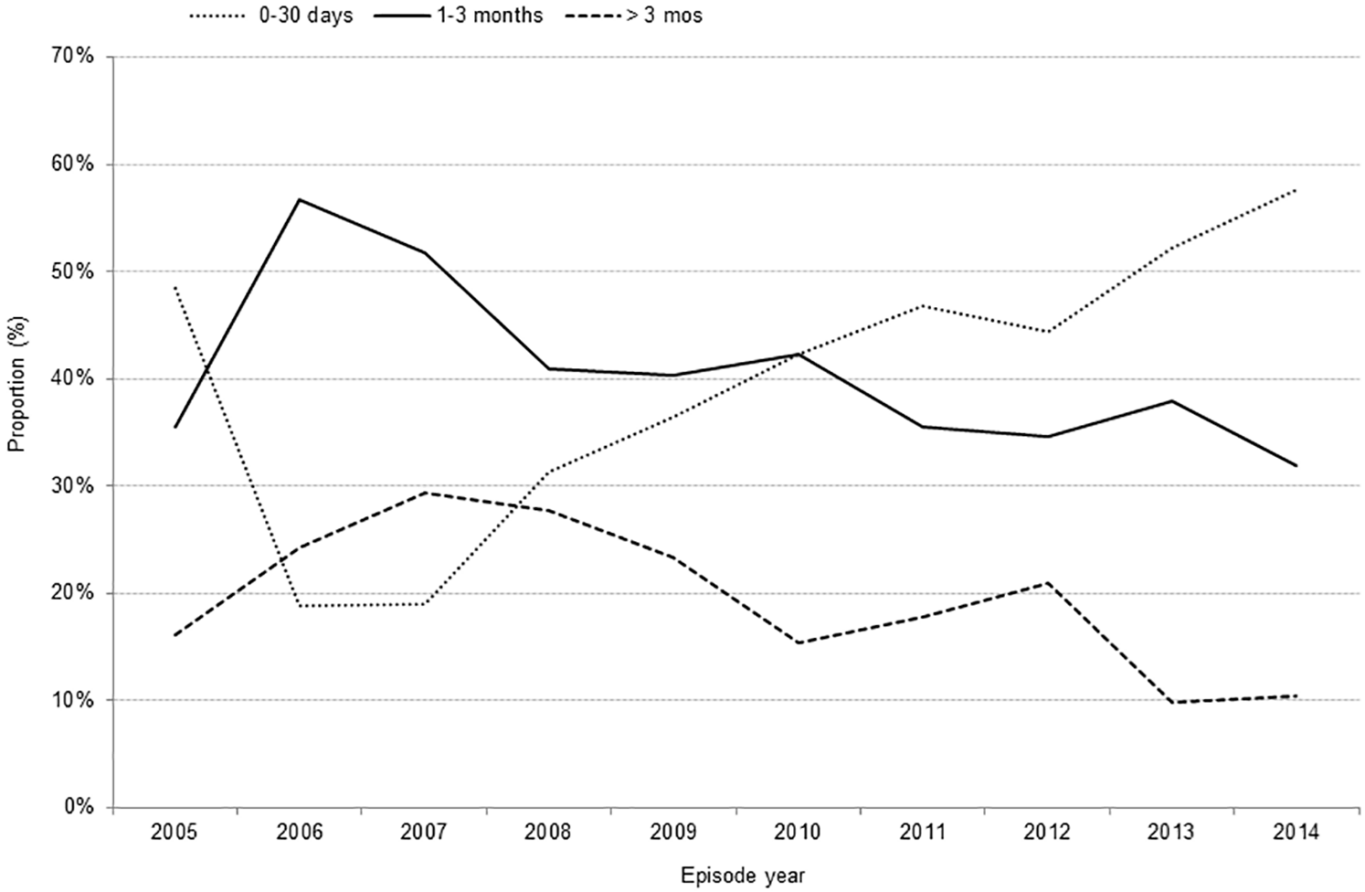
Time to public health notification of Lyme disease cases, Ontario: 2005–14 (n = 1,104)*. * Includes only cases with an onset date for symptoms reported.

Among all cases with an onset date, the proportion reported to public health ≤30 days of symptom onset increased from 2005–14 (p < 0.0001), while the proportions for cases reported between 1–3 months (p = 0.0017) and >3 months (p < 0.0001) decreased (Fig 5). Notification occurred ≤30 days of symptom onset for 45.2% of cases, within 1–3 months for 38.0% of cases, and >3 months for 16.8% of cases (Table 1). Irrespective of disease stage, earlier notification (within 30 days) occurred more frequently in Eastern Region (data not shown).

### Clinical manifestations and outcomes—All cases

No symptoms data were available for 7.9% (97/1,230) of reported LD cases. Of the remaining 1,133 cases, 69.6% reported having EM (Table 1), with the proportion of cases reporting this symptom increasing over time (p < 0.0001). Of cases with EM, 83.4% were reported as having typical EM; 20.8% with atypical EM and 4.2% with both. In 14.2% of cases, EM was the only symptom reported. Other clinical manifestations among all cases were as follows: 74.8% reported flu-like symptoms, 41.9% reported arthritic symptoms, 40.9% reported neurologic symptoms, and 5.7% reported cardiac manifestations (Table 1). Unspecified illness was reported in 2.4% of cases.

Eleven percent of cases were hospitalized (137/1,230), with the highest percent of hospitalizations occurring among cases with early disseminated disease (14.8%), followed by cases with late disseminated disease (11.0%) and cases with early localized disease (8.0%) (Table 1). Among hospitalized cases, the median age was 42.0 years, with no significant sex difference. The highest proportion of hospitalization among LD cases occurred among the 15–19 and 40–44 year age groups (data not shown).

#### Early localized disease

**Fig 6:**
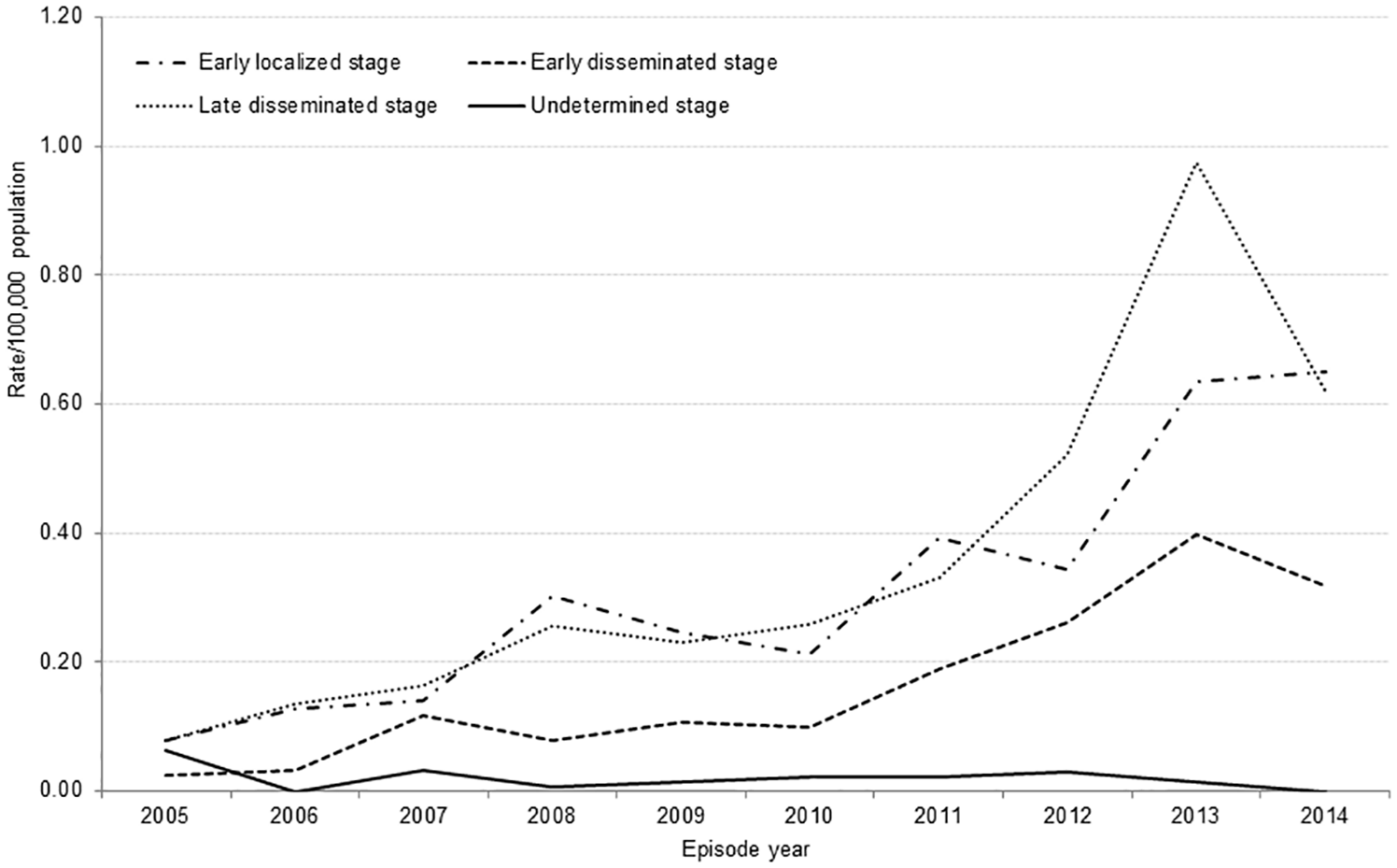
Incidence rate per 100,000 for Lyme disease cases by stage, Ontario: 2005–14 (n = 1,133)*. *Includes only cases with ≥ 1 reported symptom.

Symptoms consistent with early localized disease (S1 Table) were documented for 415 (36.6%) LD cases reported from 2005–14, with the annual proportion of total cases ranging from 29.7–47.0%. Incidence rates increased over this period (p < 0.0001, Fig 6) for cases with early localized disease. The time from symptom onset to public health notification was ≤30 days for more than half of cases (221/403; 54.8%) (Table 1). Cases resided primarily in Eastern Region (43.9%), followed by Toronto (18.8%) and Central East Region (17.6%) (Fig 3), however, the only significant rate of increase over time was observed in Eastern Region (p<0.001).

For cases with early localized disease, EM (87.2%) and flu-like symptoms (56.9%) were the primary symptoms reported. EM was the only presenting symptom for 164 (39.5%) cases whereas flu-like symptoms were the only symptoms reported for 12.8% of cases. EM was classified as typical for 309 cases (85.4%) and atypical for 69 cases (19.1%); 16 (4.4%) cases had both.

#### Early disseminated disease

Symptoms consistent with early disseminated disease (S1 Table) were reported for 216 (19.1%; range = 9.7–25.9%) LD cases over the period 2005–14. Annual incidence rates increased over the period (p < 0.0001) (Fig 6). The time from symptom onset to public health notification was ≤ 30 days for 44.3% (94/212) of cases (Table 1). The highest proportion of early disseminated cases resided in Eastern Region (41.2%) (Fig 3), and the only significant rate on of increase also occurred in this region (p<0.001).

The distribution of symptoms for the 216 cases with early disseminated disease was as follows: 204 were reported as having neurologic symptoms (94.4%), including 45 cases with Bell’s palsy; 135 cases were reported as having EM (62.5%), and 22 cases were reported as having cardiac manifestations (10.2%) (Table 1).

#### Late disseminated disease

Symptoms consistent with late disseminated disease (S1 Table) were reported for 475 LD cases (41.9%; range = 32.3–48.2%) reported from 2005–14. For these cases, the incidence rate in 2014 decreased despite an overall increasing trend for the period (p < 0.0001) (Fig 6). All 475 cases in this category had symptoms consistent with Lyme arthritis, characterized chiefly by arthralgia (440; 92.6%) and arthritis (35; 7.4%). EM was reported by 292 (61.5%) cases; neurologic manifestations by 259 (54.5%) cases; and cardiac manifestations by 43 (9.1%) cases (Table 1).

More than half of cases occurred in Eastern Region (52.8%) where the rate of increase was the only significant observation among the seven regions (p<0.001). Almost two-thirds of late disseminated cases were reported to public health >30 days after onset (Table 1).

## Discussion

### Epidemiologic trends

This is the first study to describe the clinical manifestations of LD in Ontario, Canada. The epidemiological (i.e., time to public health notification, region of residency, seasonality) and clinical (i.e., hospitalization, presentation of EM) features differed significantly between cases with early localized, early disseminated and late disseminated disease. Symptoms consistent with early localized disease were identified for 37% of cases reported from 2005–14, while symptoms consistent with early disseminated disease and late disseminated disease were reported for 19% and 42% of cases, respectively. Incidence rates for these three disease stages also increased over time but with higher rates of early localized and late disseminated diseases for all years and notable regional differences.

Available studies on blacklegged tick surveillance in Ontario and Canada provide evidence that is supportive of a true increase in the risk of infection with *B*. *burgdorferi* [14–16]. These studies show continued range expansion of the blacklegged tick population in Ontario, which is consistent with the geographic distribution of cases. This expansion is aided by warming temperatures, marked by an increase in annual cumulative degree days above 0°C. The ecological impact has been higher rates of survival and reproduction and wider dispersal and establishment of blacklegged tick populations across a broader geographic range. As with the incidence of human cases, the rate of expansion of the blacklegged tick population has been fastest in the eastern portion of the province with further growth projected northward and westward from areas of highest risk. Additional factors such as human encroachment into forested areas and forest fragmentation may have also contributed to the higher risk of exposure to LD in Ontario [17–19].

Improvements to public health surveillance and notification, as well as greater awareness of LD among the general public and health care providers may have also contributed to the observed increase in the incidence of LD. In 2009, the Ontario surveillance case definition was updated which resulted in improved sensitivity (see S1 File); additional guidance that standardized data collection and reporting were also developed for public health units managing cases of LD. In 2010, the Ministry of Health and Long-Term Care also launched a province-wide LD campaign [20]. While the impact of this awareness campaign on health seeking behaviours, clinical practices and reporting patterns has not been assessed, studies in both low and high endemic areas in the United States (U.S.) [21, 22] and Canada [23, 24] have described the relationships between provider knowledge and diagnostic and reporting patterns. These studies noted that while providers’ overall knowledge of LD was moderate to high, their understanding of diagnostic criteria was low, with approximately one-quarter of surveyed physicians correctly identifying EM as being diagnostic of LD. Providers were also more likely to accurately identify arthritis, the characteristic feature of late disseminated LD as a symptom of LD (96–99%) but less likely to accurately identify third-degree heart block (63–73%), meningitis (75–81%) and radiculoneuropathy (80–84%), which typically manifest in early disseminated disease. In British Columbia, Canada, where endemicity is low, physicians reported empirically diagnosing and treating more patients for LD than were reported to public health authorities [23]. These findings may explain in part the observed distribution of the three disease stages among LD cases in Ontario, where early and late disseminated disease occurred more frequently, accounting for almost 2/3 of LD cases that were staged.

### Clinical manifestations

The clinical features reported by cases in our study aligned with the relative proportions for cases reported via other passive surveillance systems in Nova Scotia, Canada (2002–13) and the U.S. (1992–2006)[8, 25]. EM was the most frequently reported clinical presentation, occurring in 70% of Ontario cases. In Nova Scotia, this proportion was 53% [8], and in the US this proportion was 69% [25]. Comparable rates of flu-like illnesses were also reported in Nova Scotia (70%) and among our cases (75%). Symptoms consistent with arthritic manifestations were reported more frequently (42%) in our study compared to 23% in Nova Scotia and 32% in the U.S., as were neurological symptoms (41% in Ontario compared to 13% in Nova Scotia and 12% in the U.S.), and cardiac manifestations (6% in Ontario compared to 1% in Nova Scotia and <1% in the U.S.) [5, 8, 25].

The aggregation of mainly case-reported versus clinician-reported symptoms may have contributed to the observed differences in the clinical features of the LD cases in our study. Case reported symptoms were not objectively confirmed upon collection by local health units and some of these reported symptoms, though part of the clinical spectrum for LD, may not have been related to the cases’ diagnosis. This could have resulted in misclassification into any of the three disease stages and possible over-estimation of the degree of dermatologic, cardiac, neurologic and arthritic involvement among our cases. In Nova Scotia where EM is physician diagnosed, the proportion of cases with EM was substantially lower than case reports of EM in our study and Bacon *et al’s* [8, 25]. However, the difference in the proportion of cases with flu-like symptoms was less striking in that comparably high proportions were observed for this symptom [8], which includes a range of clinical manifestations with which clinicians and the public tend to be more familiar with and therefore more likely to describe accurately.

Aside from differences arising from the geographical distribution of *Borrelia* genospecies [26–28], variations in clinical features across studies of populations where one genospecie predominates may be attributed to surveillance methods (i.e., laboratory versus physician reporting with or without case follow-up) [29] and/or case definitions used and the resultant bias towards later stage disease. This type of detection bias may have arisen in our study because clinical diagnosis of LD is most often supported by laboratory tests that perform better in later stage disease—the result being early stage disease is likely to be underestimated and later stage disease is likely to be over-represented.

### Geographical variations

In our study, we observed important geographical differences that could have impacted diagnosis and reporting to public health, and thus the distribution of early and disseminated symptoms among reported cases. For example, the highest incidence of LD occurred in health units in the Eastern, Central West and North West regions, regions in which the risk of LD has been established based on the presence of *B*. *burgdoferi* infected blacklegged tick populations. In all seven regions, the majority of cases reported symptoms compatible with disseminated disease; however, Eastern Region, an area with the longest history of endemicity in Ontario, accounted for the largest proportion of early localized infections. Additionally, earlier public health notification (within 30 days of onset) occurred more frequently in Eastern Region, while later notification (>3 months after onset) was more frequent in North East Region, which to date has no identified risk areas. This suggests that familiarity with LD may be greater in areas of higher risk, leading to earlier recognition, diagnosis and treatment based on clinical findings and appropriate ordering of diagnostic tests. Our interpretation of these results are supported by earlier findings from another Canadian study that showed that differing clinical experiences arising from the length of time under surveillance impact knowledge of EM, and intention to diagnose and treat EM according to accepted guidelines [24].

### Limitations

Under-reporting and misclassification are two of the main limitations of this study that could have resulted in biased distributions of case characteristics including their clinical manifestations. The reasons for under-reporting of LD in Ontario are likely similar to other jurisdictions that rely on passive surveillance. In the US, the under-reporting factor has been determined to be approximately 10 based on LD testing, medical claims, and physicians reporting practices [30–32]. Although, the degree of under-reporting in Ontario has not been determined, detection and reporting are assumed to be skewed towards cases confirmed by serology, which means that cases with later stage disease are more likely to be over-represented in our data. The corollary is that under-reporting is more likely for early stage LD cases where the antibody response may be insufficient to yield a positive serological test result. For these cases, physicians may be more likely to manage patients clinically but may fail to report to public health authorities. Given that the provincial public health laboratory is the only one that conducts tests for LD in Ontario and results are automatically forwarded to local public health units, under-reporting due to the non-reporting of diagnosed cases is largely restricted to cases diagnosed on the basis of clinical presentation and exposure history alone.

Other factors specific to the surveillance of LD in Ontario also affect the interpretation of these findings. It is important to emphasize that iPHIS, the reportable disease reporting system in Ontario, was not designed to provide complete data on symptoms, and there is likely variation in the collection of symptom data across the province’s 36 local health units. As a result, we could not assess whether multiple EM, one of the earliest indications of disseminated disease, occurred among cases reporting EM, or whether reported symptoms preceded or followed the exposure that resulted in infection with *B*. *burgdorferi*. Thus it is possible that some reported symptoms were unrelated to LD and that cases with unidentified multiple EM could have been misclassified as early localized infections. Secondly, our study-specific disease stages are based on symptoms reported at the time of case follow-up. Thus, progression to disseminated disease after follow-up was not captured. Despite the possibility of misclassifying symptoms and under-reporting cases, we were able to elucidate trends that have important public health and practice implications.

### Public health and practice implications

The public health and primary-care practice implications of this study stem from our chief findings: (1) early and late disseminated disease accounted for the majority of LD cases reported in Ontario from 2005–14, (2) time to public health notification for more than half of all cases occurs >30 days after symptom onset, and (3) regional variations are reflected in the epidemiological and clinical features of cases. From public health and practice standpoints, several factors could have contributed to this pattern of disease, including the complexity and non-specificity with which LD presents, the fact that EM is poorly recognized as being diagnostic [21, 23], and that EM is not reported or observed for a large proportion of cases (≈40% of cases with disseminated disease in Ontario). These findings demonstrate the need for improvements in the diagnosis of early localized disease and could serve as important benchmarks of Ontario’s progress towards earlier diagnosis and treatment under the recently implemented LD reduction strategy—*Combatting Lyme Disease Through Collaborative Action*: *Ontario’s 10-Step Education and Awareness Plan* [33]. Public health efforts to reduce the overall burden of LD in Ontario should continue to pursue opportunities to educate primary care physicians on the full clinical spectrum of LD with the goal of increasing earlier diagnosis, reducing the incidence of later stage disease, and improving the reporting of clinically diagnosed cases. However, when developing clinician educational outreach activities, it is important to recognize the variation in learning and/or practice needs that likely exist for clinicians working in established endemic, recently endemic and non-endemic areas. For the public, educational efforts should also acknowledge regional differences, focusing on education that underscores the importance of prevention and early symptoms recognition and the need for timely treatment following the onset of symptoms. To support these strategies, new studies are required to assess healthcare provider knowledge, beliefs and practices (diagnostic and treatment), as well as public knowledge, attitude and behaviours with respect to LD. Epidemiologic studies that utilize chart reviews of physician diagnoses of Lyme carditis, Lyme arthritis or neuroborreliosis, and temporally situate these symptoms in relation to the case’s exposure to blacklegged ticks may also be required to more precisely elucidate the clinical manifestations of LD in Ontario.

## Conclusions

Despite possible misclassification of the three clinical stages of LD, we were able to elucidate trends. Incidence rates for the three clinical stages of LD increased over time, with the relative proportion of early to late disseminated disease varying spatially across Ontario. The proportion of cases reported to public health within 30 days of illness onset also increased and the proportion reported >3 months after illness onset decreased. Of importance are our findings of higher occurrence of early localized disease and earlier public health notification for regions with well-established or recently established risk areas for LD, and of higher occurrence of later public health notification for regions with no identified risk areas or recently established risk areas. These findings of increasing risk and regional variation in the incidence of LD demonstrates the relevance of targeted measures to increase awareness, promote earlier recognition and encourage greater uptake of preventive measures by the general public. For healthcare professionals, strategies aimed at improving earlier recognition, diagnosis and treatment based on presenting symptoms and exposure histories, and not surveillance case definitions, should also be emphasized. With increasing awareness and familiarity with LD, we expect decreases in exposures, as well as earlier recognition, diagnosis and treatment, which would result in a reduction in overall incidence and a shift in the ratio of disseminated to early localized disease. However, maximizing gains toward these goals must consider the implementation of different strategies for different regions based on the presence of risk that is well established, recently established or not yet identified, as well as on the length of time that an area has been identified as being higher risk for LD.

## Supporting information

S1 File Ontario Lyme disease case definitions: 2005–14. (PDF)

S1 Table Reported symptoms for Lyme disease cases grouped by syndrome and stage, Ontario: 2005–14 (n = 1,133) *. *Includes all cases that report ≥ 1 symptom.
(PDF)
