# Supplementary material for: Clinical manifestations of reported Lyme disease cases in Ontario, Canada: 2005–2014
Source: PLoS One. 2018 Jun 1;13(6):e0198509. doi: 10.1371/journal.pone.0198509 (PMC5983483; doi:10.1371/journal.pone.0198509)
Supplement: S1 File — (PDF) [file pone.0198509.s001.pdf]

S1. Appendix Ontario Lyme disease case definitions: 2005–14.

2005–2008

Confirmed case

Endemic

(a) Isolation of *Borrelia burgdorferi* from a clinical specimen **OR**

(b) Erythema migrans observed by a physician **OR**

(c) At least one clinically compatible late manifestation (neurologic, cardiac or musculoskeletal) **AND** laboratory evidence of *B. burgdorferi* infection

Non-endemic

(d) Erythema migrans observed by a physician and laboratory evidence of *B. burgdorferi* infection

Probable Case

Endemic

(a) Physician recognition of erythema migrans as reported by patient

Non-endemic

(b) Compatible late manifestation (neurologic, cardiac or musculoskeletal) **AND** laboratory evidence for *B. burgdorferi* infection

2009–2014

Confirmed case

(a) Erythema migrans (EM) with laboratory confirmation by polymerase chain reaction (PCR) or culture

OR

(b) EM with laboratory support by serological methods, and a history of residence in, or visit to, an endemic area **OR**

(c) Objective symptoms of disseminated Lyme disease with laboratory confirmation by PCR or culture

OR

(d) Objective symptoms of disseminated Lyme disease with laboratory support by serological methods, **AND** a history of residence in, or visit to, an endemic area

Probable case

(a) EM with laboratory support by serological methods but with no history of residence in, or visit to, an endemic area **OR**

(b) Objective symptoms of disseminated Lyme disease with laboratory support by serological methods, but with no history of residence in, or visit to an endemic area **OR**

(c) EM without laboratory confirmation, but with history of residence in, or visit to, an endemic area.
