# Supplementary material for: Clinical manifestations of reported Lyme disease cases in Ontario, Canada: 2005–2014
Source: PLoS One. 2018 Jun 1;13(6):e0198509. doi: 10.1371/journal.pone.0198509 (PMC5983483; doi:10.1371/journal.pone.0198509)
Supplement: S1 Table — *Includes all cases that report ≥ 1 symptom. (PDF) [file pone.0198509.s002.pdf]

S1 Table. Reported symptoms for Lyme disease cases grouped by syndrome and stage, Ontario: 2005–14 (n = 1,133).[*]

**Reported symptoms**		**Number of cases**
**Early localized disease**		
EM, classic	Erythema migrans [EM]	640
	Rash, "bull's eye" / EM ≥ 5 cm diameter	14
	Erythema	4
EM, atypical	Swelling, localized	22
	Rash, other	14
	Cellulitis	7
	Rash, not described	6
	Rash, itchy	5
	Rash, macular	4
	Rash, maculopapular	4
	Rash on body, desquamation not present	2
	Rash, papular	2
	Rash	1
	Rash, centrifugal distribution	1
	Rash, rose spots	1
Flu-like symptoms	Fatigue	600
	Headache	496
	Fever	484
	Myalgia [muscle pain]	426
	Chills	57
	Sweating, at night	14
	Sweating	13
	Back pain	12
	Weak	11
	Body, generalized aches	7
	Malaise [general unwell feeling]	7
	Lethargy	6
	Muscle weakness	4
	Back ache	2
	Cervical lymphadenopathy	1
	Fever, cycling	1

	Sweating, profuse [diaphoresis]	1
**Early disseminated disease**		
Bell's Palsy	Bell's palsy / other cranial neuritis	79
	Facial paralysis [bell's palsy]	2
Other neurological symptoms	Neck pain	260
	Paresthesia [tingling, numbness or burning]	135
	Cognitive impairment or mood disturbances	87
	Visual symptoms	78
	Auditory symptoms	32
	Dizziness	27
	Lymphocytic meningitis/encephalitis/encephalomyelitis	13
	Radiculoneuropathy	11
	Neck, stiff	8
	Insomnia	4
	Photophobia	4
	Memory loss	3
	Vision, blurred/double	3
	Muscle paralysis	2
	Neurological deficits	2
	Seizures	2
	Sore throat/hoarseness/difficulty swallowing	2
	Swallowing difficulty [dysphagia]	2
	Agitation or restlessness	1
	Altered mental status	1
	Confusion	1
	Convulsions	1
	Hearing impairment	1
	Hearing loss	1
	Irritability	1
	Meningitis	1
	Mouth/tongue, tingling/burning sensations	1
	Neurological involvement	1
	Neurological symptoms	1
	Paralysis, partial [paresis]	1
	Swallowing pain	1
	Confusion/forgetful	0
Cardiac symptoms	Palpitations / arrhythmia	49
	Chest pain	6
	A-V heart block [second or third degree]	5
	Shortness of breath	3
	Heart flutter [palpitations]	1
	Heart involvement	1
	Heart rate increase [tachycardia]	1

Late disseminated disease		
Lyme arthritis	Arthralgia [joint pain]	440
	Joint inflammation [arthritis]	2
	Joint inflammation, reactive [reactive arthritis]	1
	Limb pain	1
**Symptoms not related to Lyme disease**		
Symptoms not consistent with the defined LD stages	Vomiting	17
	Anorexia [loss of appetite]	14
	Abdominal pain	6
	Jaw pain	6
	Cough	5
	Coryza [runny nose]	4
	Dehydration	3
	Eye, pain	3
	Nausea [in children]	3
	Rash, painful	3
	Abdominal bloating or flatulence	2
	Breathing stops/suspends [apnea]	2
	Constipation	2
	Liver function abnormality	2
	Loss of weight	2
	Nausea	2
	Throat, sore	2
	Urine, dark	2
	Abscesses, external	1
	Cough, dry	1
	Coughing with apnea/vomiting	1
	Cough, not productive	1
	Enlarged liver and spleen [hepatosplenomegaly]	1
	Eyelid swelling [periorbital edema]	1
	GI symptoms, general	1
	Hair loss [alopecia]	1
	Infection, localized	1
	Joint inflammation, septic [septic arthritis]	1
	Palms/hands, redness [palmar erythema]	1
	Rash, petechiae	1
	Septicemia	1
	Skin, itchy	1
	Skin/wound redness, increasing quickly	1
	Tonsillitis	1
	Tonsillitis, exudative	1
	Lymphadenopathy	59
	Diarrhea	4
	Cramps	2

	Fainting	1
	Leukopenia [low white blood cell count]	1
	Muscle spasms	1
	Other [specify]	1
	Skin/muscle, extreme pain to touch	1

*Includes all cases that reported ≥ 1 symptom.
